# Role of Volatiles from the Endophytic Fungus *Trichoderma asperelloides* PSU-P1 in Biocontrol Potential and in Promoting the Plant Growth of *Arabidopsis thaliana*

**DOI:** 10.3390/jof6040341

**Published:** 2020-12-06

**Authors:** Nongnat Phoka, Nakarin Suwannarach, Saisamorn Lumyong, Shin-ichi Ito, Kenji Matsui, Siwaret Arikit, Anurag Sunpapao

**Affiliations:** 1 Ratchaburi Campus, King Mongkut’s University of Technology Thonburi, Ratchaburi 70150, Thailand; nongene@gmail.com; 2 Department of Biology, Faculty of Science, Chiang Mai University, Chiang Mai 50200, Thailand; suwan.462@gmail.com (N.S.); saisamorn.l@cmu.ac.th (S.L.); 3 Research Center of Microbial Diversity and Sustainable Utilization, Chiang Mai University, Chiang Mai 50200, Thailand; 4 Department of Biological and Environmental Sciences, Graduate School of Science and Technology for Innovation, Yamaguchi University, Yamaguchi 753-8515, Japan; shinsan@yamaguchi-u.ac.jp; 5 Research Center for Thermotolerant Microbial Resources (RCTMR), Yamaguchi University, Yamaguchi 753-8515, Japan; 6 Department of Biological Chemistry, Graduate School of Science and Technology for Innovation, Yamaguchi University, Yamaguchi 753-8515, Japan; matsui@yamaguchi-u.ac.jp; 7 Department of Agronomy, Faculty of Agriculture at Kamphaeng Saen, Kasetsart University Kamphaeng Saen Campus, Nakhon Pathom 73140, Thailand; arikit@gmail.com; 8 Agricultural Innovation and Management Division, Faculty of Natural Resources, Prince of Songkla University, Hatyai, Songkhla 90112, Thailand

**Keywords:** *Arabidopsis*, biocontrol agent, volatile compounds, *Trichoderma*

## Abstract

Fungal volatile organic compounds (VOCs) emitted by *Trichoderma* species interact with a plant host and display multifaceted mechanisms. In this study, we investigated the antifungal activity of VOCs emitted by *Trichoderma asperelloides* PSU-P1 against fungal pathogens, as well as the ability of VOCs to activate defense responses and to promote plant growth in *Arabidopsis thaliana*. The strain’s VOCs had remarkable antifungal activity against fungal pathogens, with an inhibition range of 15.92–84.95% in a volatile antifungal bioassay. The VOCs of *T. asperelloides* PSU-P1 promoted the plant growth of *A. thaliana*, thereby increasing the fresh weight, root length, and chlorophyll content in the VOC-treated *A. thaliana* relative to those of the control. High expression levels of the chitinase (*CHI*) and β-1,3-glucanase (*GLU*) genes were found in the VOC-treated *A. thaliana* by quantitative reverse transcription polymerase chain reaction (RT-PCR). The VOC-treated *A. thaliana* had higher defense-related enzyme (peroxidase (*POD*)) and cell wall-degrading enzyme (chitinase and β-1,3-glucanase) activity than in the control. The headspace VOCs produced by PSU-P1, trapped with solid phase microextraction, and tentatively identified by gas chromatography–mass spectrometry, included 2-methyl-1-butanol, 2-pentylfuran, acetic acid, and 6-pentyl-2H-pyran-2-one (6-PP). The results suggest that *T. asperelloides* PSU-P1 emits VOCs responsible for antifungal activity, for promoting plant growth, and for inducing defense responses in *A. thaliana*.

## 1. Introduction

*Trichoderma* species are widely considered potential biological control agents (BCAs) against several plant diseases due to their unique characteristics. For instance, they can produce bioactive secondary metabolites [1], compete for nutrients and space [2,3], induce plant defensive mechanisms [2,4,5], and promote plant growth [6]. They also feature mechanisms such as antibiosis by producing extracellular cell wall-degrading enzymes [3,7] and mycoparasitism [8]. Such abilities allow to use *Trichoderma* species as BCAs in agriculture for controlling plant diseases and for promoting plant growth [9].

*Trichoderma* species have been reported as endophytes due to their ability to colonize internal plant tissues. For instance, *Trichoderma* species have been shown to persist within a plant’s roots and in above-ground tissues through endophytic associations [10]. Endophytes are known as sources of novel biological benefits to their host plants [11]. The combination of antibiosis, a widespread mechanism used by *Trichoderma* species, and the endophytic relationship plays an important role in defense against several pathogens by releasing metabolites that act as antifungal compounds [1].

The metabolites known as volatile organic compounds (VOCs) released from endophytic *Trichoderma* species are of high interest, and may benefit the host plant by contributing additional defenses against pathogens [2,4,12]. The properties of VOCs include a low boiling point, a low molecular mass, and a high vapor pressure [13]. Such ability to produce VOCs is found in plants [14] and in microorganisms [15]. Recent publications have shown that the VOCs released by *Trichoderma* display antifungal activities [1,4,6] and promote plant growth [16].

*Trichoderma asperelloides* has been used as a biocontrol agent of *Sclerotinia sclerotiorum* [17]. However, the ability of the VOCs emitted by *T. asperelloides* PSU-P1 to contribute to antifungal activity and to promote plant growth has not yet been clarified. Therefore, this research aimed to demonstrate that the VOCs of *T. asperelloides* PSU-P1 are major factors in the antifungal activity against fungal disease pathogens and in the promotion of the growth of the plant model *Arabidopsis thaliana*.

## 2. Materials and Methods

### 2.1. Trichoderma Species and Pathogen Sources

The endophytic fungus *T. asperelloides* PSU-P1 [18] was used in this study. Fungal pathogens causing emerging diseases that have recently been reported in Thailand were obtained from the Culture Collection of Pest Management, Faculty of Natural Resources, Prince of Songkla University, Thailand. A list of the microorganisms used in this study is provided in Table 1. *T. asperelloides* PSU-P1 and the pathogens were cultured on potato dextrose agar (PDA) (HiMedia, Mumbai, India) for 3 days at 28 ± 2 °C before use in this study.

### 2.2. Volatile Antifungal Bioassay

**Figure 1:**
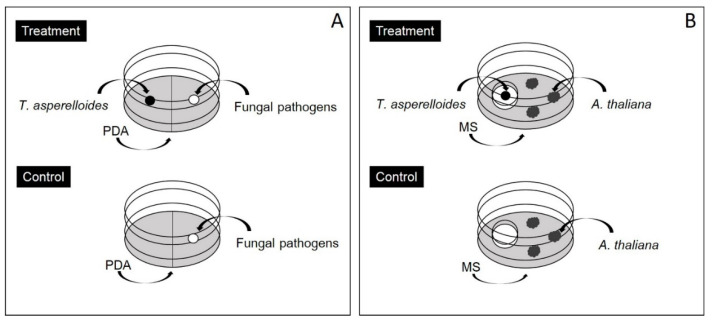
Overview of the experimental setup and the effect of volatile organic compounds (VOCs) on fungal growth, induced defense responses, and increased growth. A schematic overview of the plate-within-a-plate system for fungal growth (**A**) and for plant growth (**B**). PDA, potato dextrose agar; MS, Murashike and Skoog medium.

The antifungal activity of the volatiles emitted by *T. asperelloides* PSU-P1 was assessed by using a volatile antifungal bioassay, as previously described by Dennis and Webster [25] with some modifications. *T. asperelloides* PSU-P1 and the pathogens were grown on PDA for 5 days. The study was performed in two-room plates, 9 cm in diameter (BIOMED, Bangkok, Thailand). The plates had agar plugs (0.5 cm in diameter) cut from stock cultures placed on one side of culture plate, whereas the other side had agar plugs of pathogens (Figure 1A). The plates were taped with parafilm and incubated at 28 ± 2 °C for 5 days. For the control cases, an agar plug of each pathogen was placed on PDA on one side of culture plate, while the other side contained PDA alone. The experiments were set up in triplicate and repeated twice. The fungal growth of tested fungi was measured as colony diameters and the percentage of inhibition was calculated by:

(1) $$\mathrm{Percent}\;\mathrm{inhibition}(\%)=\frac{Dc-Dt}{Dc}\times 100,$$

where *Dc* is the colony diameter of the pathogen in the control and *Dt* is the colony diameter of the pathogen in the treatment [26].

### 2.3. Plant and Growth Conditions

*A. thaliana* ecotype Columbia seeds were surface-disinfected with 70% ethyl alcohol and 0.5% sodium hypochlorite (NaOCl). The seeds were rinsed with sterile distilled water (DW) to remove excess NaOCl and air-dried on sterile paper in a laminar air flow cabinet. A total of eight seeds were placed on Murashike and Skoog (MS) medium (HiMedia, Mumbai, India) [27] in a plastic 9 cm Petri dish. The cell culture plates were then incubated in a TOMY CLE-303 Cultivation Chamber (TOMY Seiko, Tokyo, Japan) maintained at 60 ± 2% relative humidity (RH) and 22 ± 2 °C with a light/dark photoperiod of 16:8 for 14 days.

### 2.4. Volatile Exposure Bioassay

To test the effect of *Trichoderma* VOCs on *A. thaliana* growth, an experiment was conducted using cell culture plates (BIOMED, Bangkok, Thailand), as shown in Figure 1B. The outside culture plate contained MS medium for culturing *A. thaliana* seeds (8 seeds), whereas the inside culture plate contained PDA for culturing *T. asperelloides* PSU-P1. Both the *A. thaliana* seeds and the *T. asperelloides* PSU-P1 were grown in a shared atmosphere on the culture plate and incubated in a growth chamber as described in Section 2.3. The wells without *Trichoderma* inoculation (PDA alone) served as the control. The experiments were set up in triplicate and repeated twice. At the end of the VOC exposure periods, the *A. thaliana* plants were removed from the exposure conditions and subjected to phenotypic characterization (fresh weight and root length) and to enzyme assays.

### 2.5. Plant Chlorophyll Measurements

Total chlorophyll (Chl) content was measured according to the method previously described by Moran [28] and Kaewsuksaeng et al. [29] with some modifications. Whole plant tissues of *A. thaliana* were subjected to total chlorophyll extraction. The shoots of *A. thaliana* (0.5 g) were submerged in *N*,*N*-dimethylformamide overnight at 4 °C. The total chlorophyll content in the extracts was determined using a UV-2000 Spectrophotometer (Hitachi, Tokyo, Japan). The total chlorophyll content was calculated as:

Chl a (µg mL^−1^) = 12.64 OD_664_ − 2.99 OD_647_, (2)

Chl b (µg mL^−1^) = −5.60 OD_664_ + 23.26 A_647_, (3)

Chl a (mg 100 g FW) = Chl a (µg mL^−1^) × 20.5 × 100/0.5 × 1/1000, (4)

Chl b (mg 100 g FW) = Chl b (µg mL^−1^) × 20.5 × 100/0.5 × 1/1000, (5)

where A_664_ is the absorbance at 664 nm and A_647_ is the absorbance at 647 nm.

### 2.6. RNA Extraction and Quantitative Reverse Transcription PCR Analysis

After 3 days of the VOC treatment, whole *Arabidopsis* from 20 plants were subjected to RNA extraction by Trizol reagent (ThermoFisher, Waltham, MA, USA) according to the manufacturer’s instructions. The total RNA was collected, air-dried, and dissolved in RNase-free DW Approximately 1 μg of total RNA was reverse-transcribed to single-strand cDNA and subjected to quantitative reverse transcription PCR (qRT-PCR) as previously described by Dumhai et al. [30]. The qRT-PCR reactions were performed using the iScript One-Step RT-PCR reagent with SYBR Green (Bio-Rad, Hercules, CA, USA) containing 1 ng of total RNA as the template. The actin gene (*ACT*) forward primer was used as an internal reference gene to normalize the variations of input total cDNA templates between the control and VOC-treated samples. The gene-specific primers of chitinase (*CHI*), β-1,3-glucanase (*GLU*), and peroxidase (*POD*) used in this study are shown in Table 2. The relative gene expression was analyzed by Bio-Rad CFX Manager analysis software (Bio-Rad, Hercules, CA, USA) to determine fold changes in expression relative to actin as a control.

### 2.7. Enzyme Assay

We hypothesized that plants exposed to fungal VOCs may have activated defense responses, so the enzyme activities of the VOC-treated *A. thaliana* were compared to those of the untreated plants (control). Crude protein extraction from the VOC-treated and untreated plants was conducted with potassium phosphate buffer (KPB) at pH 6.0 for peroxidase [3] and at pH 7.0 for chitinase and β-1,3-glucanase assays [31]. The extracted *A. thaliana* plants were then centrifuged at 14,000× *g* for 20 min at 4 °C, and the supernatants were then collected and used immediately for the enzyme assays.

The activity of peroxidase was determined following Vetter et al. [32] with some modifications. O-phenylenediamine (OPDA) at a concentration of 1% was used as the substrate in the peroxidase assay. After adding 0.1 mL of 0.3% H_2_O_2_ for 10 min, the increase in absorbance at 430 nm was measured with a UV/VIS spectrophotometer UV5300 (METASH, Shanghai, China) and the peroxidase activity is expressed as ΔA 430 U mL^−1^. The chitinase and β-1,3-glucanase activities were determined using the 3,5-dinitrosalicylic acid (DNS) method [33]. Reaction mixtures containing colloidal chitin or laminarin (Sigma Aldrich, Saint Louis, MO, USA) were used as the substrate in the chitinase or β-1,3-glucanase assay, respectively. The reducing sugar released in the test reaction mixtures was measured with the UV5300 UV/VIS spectrophotometer at 550 nm and at 575 nm for β-1,3-glucanase and chitinase, respectively. Each enzyme was assayed in three replicates from two repeats.

### 2.8. Gas Chromatography-Mass Spectrometryanalysis

*T. asperelloides* PSU-P1 was cultured on PDA incubated at 28 ± 2 °C for 5 days. Solid phase microextraction (SPME) was conducted to collect the VOCs produced by *T. asperelloides* PSU-P1 according to the method previously described by Suwannarach et al. [34]. SPME fiber was exposed to the vapor phase above *T. asperelloides* PSU-P1 for 45 min in a culture tube. Then, the adsorbent fiber was inserted into the injection port of the gas chromatograph GC 2010 (Shimadzu, Kyoto, Japan) equipped with a DB-Wax capillary column (0.25 mm × 30 m I.D., 0.25 µm in film thickness; Supelco, Sigma Aldrich, Saint Louis, MO, USA). The column temperature was programmed at an initial temperature of 40 °C for 2 min, and was then increased at a rate of 5 °C min^−1^ to a final temperature of 200 °C. Purified helium was used as the carrier gas at an initial column head pressure of 60 kPa. The fiber was desorbed at 250 °C for 57 min under a flow of helium gas prior to trapping the VOCs. A 30 s injection time was used to introduce the adsorbed VOCs into the gas chromatograph (GC) interfaced with a mass spectrometer, i.e., MS-QP2010 (Shimadzu, Kyoto, Japan). The mass spectrometer was operated at unit mass resolution. Data acquisition and data processing were conducted by the software system. The VOCs produced by *T. asperelloides* PSU-P1 were tentatively identified through computer searches of the National Institute of Standards and Technology (NIST, v17, 2014) Mass Spectral Library Search Chromatogram.

### 2.9. Effect of Commercial VOCs on the Antifungal Activity, Defense Responses, and Growth of A. thaliana

Each commercial volatile compound, namely, acetic acid, 2-methyl-1-butanol (Sigma Aldrich, Saint Louis, MO, USA), 2-pentylfuran (Sigma Aldrich, Saint Louis, MO, USA), and 6-pentyl-2H-pyran-2-one (6-PP) (Sigma Aldrich, Saint Louis, MO, USA), was used for the antifungal bioassay, enzyme assay, and phenotypic measurements. Each volatile compound was diluted as 10^−3^ (*v*/*v* or *w*/*v*) and 10 µL was applied in a sterile cotton pad [4,35] exposed to *A. thaliana* instead of culturing *T. asperelloides* PSU-P1, similar to the method in Section 2.4. The phenotypic characteristics were measured according to the method in Section 2.4. An enzyme assay was conducted to verify the enzyme activity between the control and treatment, as shown in Section 2.7.

### 2.10. Statistical Analysis

The results on the antifungal activity, chlorophyll content, enzyme activity, and plant growth were subjected to one-way analysis of variance (ANOVA). Tukey’s test and Student’s *t*-test were used to determine statistically significant differences between the treated samples and untreated control [36].

## 3. Results

### 3.1. VOCs Emitted by T. asperelloides PSU-P1 Inhibit Fungal Growth

**Figure 2:**
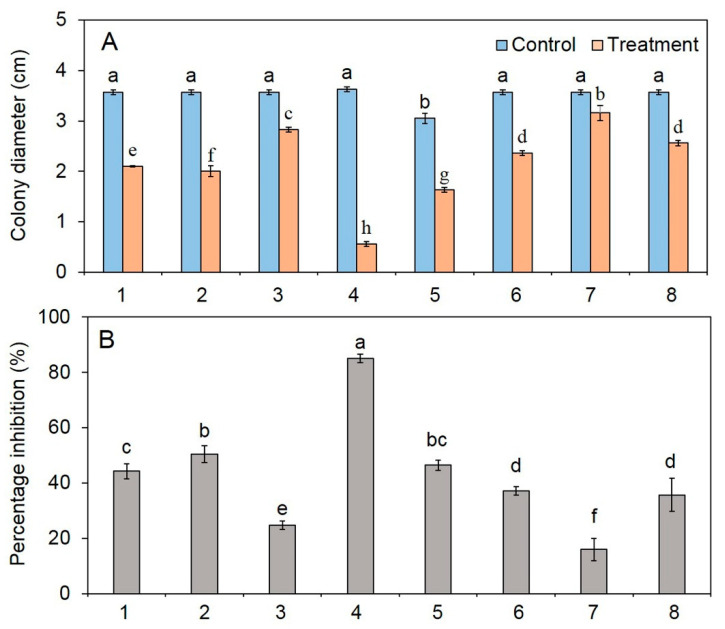
Colony diameter (**A**) and percent inhibition (**B**) by the VOCs emitted from *T. asperelloides* PSU-P1 against *Colletotrichum* sp. (1), *C. cassiicola* (2), *C. lunata* (3), *Ganoderma* sp. (4), *P. oxalicum* (5), *N. clavispora* (6), *S. rolfsii* (7), and *S. cucurbitacearum* (8). Different letters indicate statistically significant differences among treatments (*p* < 0.05) using the Tukey’s test.

After incubating the test plates of fungal pathogens (Figure 2) with the VOCs emitted from *T. asperelloides* PSU-P1 for four days, the colony diameters of all fungal growth in the test plates were significantly smaller than those of control plates (*p* < 0.05). The percent inhibition of the fungal pathogens ranged from 15.92% to 84.95% (Figure 2). The results show that the VOCs of *T. asperelloides* PSU-P1 inhibited the fungal growth of *Ganoderma* sp. with the highest percent inhibition (84.95%), whereas the lowest inhibition was found for *S. rolfsii* (15.92%).

### 3.2. VOCs Emitted by T. asperelloides PSU-P1 Increase Growth in A. thaliana

**Figure 3:**
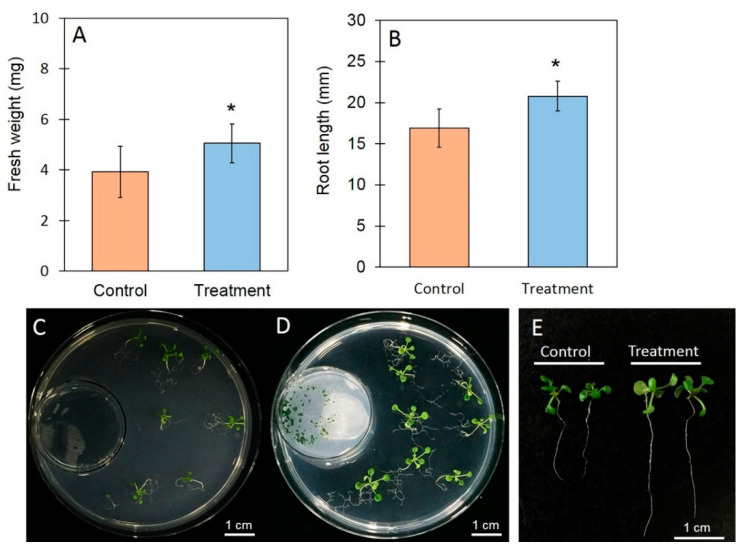
Phenotypic characteristics of *Arabidopsis thaliana*: Fresh weight (**A**); root length (**B**); control *A. thaliana* exposed to plain PDA (**C**,**E**); *A. thaliana* exposed to *T. asperelloides* PSU-P1 VOCs (**D**,**E**). Asterisks indicate statistically significant differences (* *p* < 0.05) using the Student’s *t*-test.

**Figure 4:**
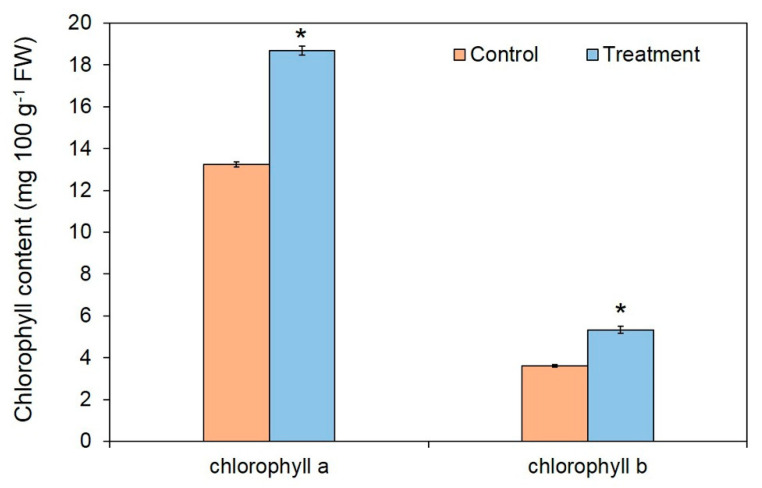
Chlorophyll *a* and *b* contents of *A. thaliana* exposed to *T. asperelloides* PSU-P1 VOCs and without VOCs. Asterisks indicate statistically significant differences (* *p* < 0.05) using the Student’s *t*-test.

After seven days of exposure, the *A. thaliana* plants were collected for measurement of fresh weight, root length, and total chlorophyll content. In the presence of the VOCs from *T. asperelloides* PSU-P1, there were significant increases in fresh weight, root length, and total chlorophyll content (Figure 3 and Figure 4). The fresh weight of control and treated plants was 3.93 ± 1.01 mg and 5.05 ± 1.15 mg, respectively (Figure 3A). The root length of control and treated plants was 16.90 ± 2.33 mm and 20.80 ± 1.81 mm, respectively (Figure 3B,D). The VOCs of *T. asperelloides* PSU-P1 also increased the total chlorophyll content (Figure 4). Chlorophyll *a* was 13.24 ± 0.11 mg g^−1^ fresh weight (FW) for the control plants and 18.68 ± 0.06 mg g^−1^ FW for the treated plants. Chlorophyll *b* content was 3.61 ± 0.21 mg g^−1^ FW for the control plants and 5.33 ± 0.14 mg g^−1^ FW for the treated plants.

### 3.3. VOCs Induce Gene Expression of Defense-Related Genes

**Figure 5:**
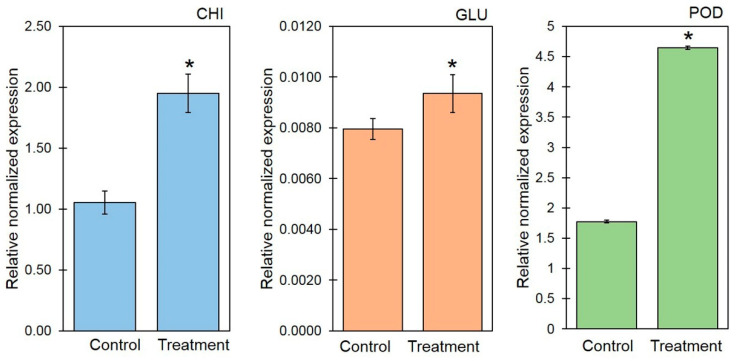
Relative gene expressions of chitinase (*CHI*), β-1,3-glucanase (*GLU*), and peroxidase (*POD*) in the VOC-treated *A. thaliana* and control. Asterisks indicate statistically significant differences (* *p* < 0.05) using the Student’s *t*-test.

To examine the role of VOCs in inducing defense responses in *A. thaliana*, the expressions of defense-related genes (*CHI*, *GLU*, and *POD*) were analyzed by qRT-PCR (Figure 5). The expression levels of the VOC-inducible genes *CHI*, *GLU*, and *POD* were significantly higher in the VOC-treated *A. thaliana* than in the control (Figure 5). The expression levels of *CHI* were 1.9495 and 1.0546 for the VOC-treated *A. thaliana* and control, respectively. The expression levels of *GLU* were 0.0094 and 0.0080 for the VOC-treated *A. thaliana* and control, respectively. The expression of *POD* in the VOC-treated *A. thaliana* was 4.6449, also higher than that of the control (1.7750).

### 3.4. Induction of Defense-Related Enzymes and Enzyme Activities by Fungal VOCs

**Figure 6:**
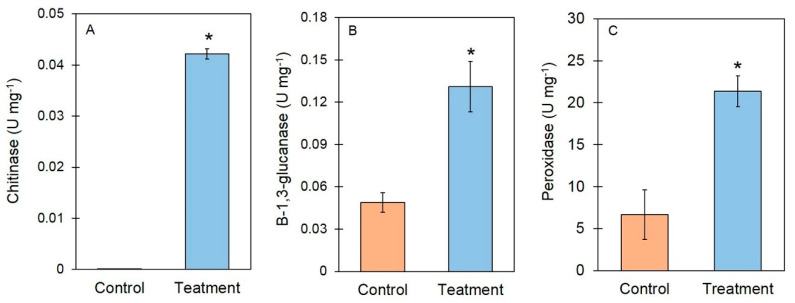
Enzyme activities of chitinase (**A**), β-1,3-glucanase (**B**), and peroxidase (**C**) between the control and VOC-treated *A*. *thaliana*. Asterisks indicate statistically significant differences (* *p* < 0.05) using the Student’s *t*-test.

The enzymes assays showed that the fungal VOCs induced the activities of the defense-related enzymes (peroxidase) and cell wall-degrading enzymes (chitinase and β-1,3-glucanase) in the treated *A. thaliana* (Figure 6). The activity of *POD* was 6.67 ± 2.49 U mg^−1^ and 23.36 ± 1.82 U mg^−1^ for untreated and VOC-treated *A. thaliana*, respectively (Figure 6). The chitinase activity was 0.001 ± 0.00 U mg^−1^ and 0.42 ± 0.014 U mg^−1^ for the untreated and VOC-treated *A. thaliana*, respectively. Meanwhile, the β-1,3-glucanase activity was 0.131 ± 0.018 U mg^−1^ and 0.49 ± 0.007 U mg^−1^ for the untreated and VOC-treated *A. thaliana*, respectively (Figure 6).

### 3.5. Identification of VOCs through SPME GC/MS

**Figure 7:**
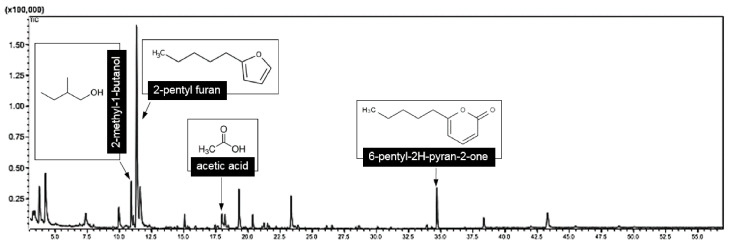
A chromatogram of the VOCs emitted by *T. asperelloides* PSU-P1 analyzed through SPME GC/MS.

Based on the SPME GC/MS analysis, the VOCs emitted by *T. asperelloides* PSU-P1 were tentatively identified as four compounds based on similarities greater than 95%, namely, 2-methyl-1-butanol, 2-pentyl furan, acetic acid, and 6-pentyl-2H-pyran-2-one. The results are summarized in Table 3, representing the VOCs produced by *T. asperelloides* PSU-P1 that were responsible for inhibiting fungal growth and for promoting growth in *A. thaliana*. The carbon counts (C) of the compounds ranged from C2 (acetic acid) to C10 (6-pentyl-2H-pyran-2-one (6-PP)). The 2-pentyl furan was dominant, contributing 30.59%, followed by 2-methyl-1-butanol (6.96%) and 6-PP (5.45%). Figure 7 shows the mass spectrum of the four compounds produced by *T. asperelloides* PSU-P1.

### 3.6. Effects of Commercial Volatile Compounds on Antifungal Activity, Induced Enzyme Activity, and Plant Phenotype

We tested the effects of each volatile compound on the fungal growth of plant pathogens, as shown in Table 4. Acetic acid could only inhibit the fungal growth of *Ganoderma* sp. The 2-pentylfuran and 2-methyl-1-butanol were found to inhibit the fungal growth of both *Ganoderma* sp. and *Penicillium oxalicum*. Moreover, 6-PP was found to show antifungal activity against *Ganoderma* sp., *P. oxalicum*, *S. rolfsii,* and *Stagonosporopsis cucurbitacearum* (Table 4).

**Figure 8:**
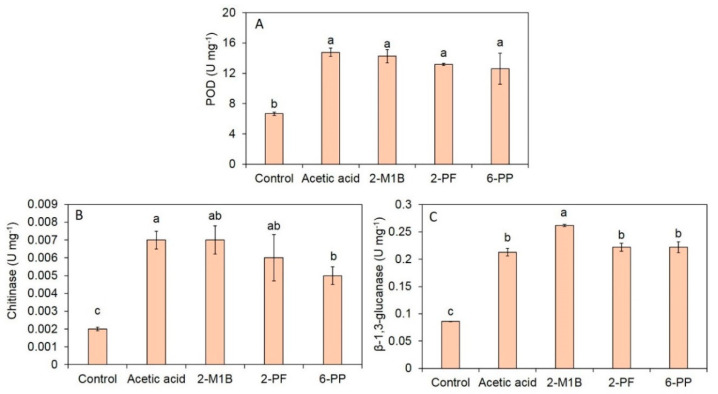
Activities of the defense-related enzymes peroxidase (**A**) and cell wall-degrading enzymes chitinase (**B**) and β-1,3-glucanase (**C**) between the control and VOC-treated *A*. *thaliana.* Different letters indicate statistically significant differences among treatments (*p* < 0.05) using the Tukey’s test.

The effects of each commercial volatile compound on the induced defense responses in *A. thaliana* were assessed using enzyme assays (Figure 8). The results show that the activity of *POD* of *A. thaliana* exposed to acetic acid, 2-methyl-1-butanol, 2-pentylfuran, and 6-PP was 14.76, 14.27, 13.19, and 12.62 U mL^−1^, respectively, significantly higher than in control (6.66 U mL^−1^). The chitinase activity of *A. thaliana* exposed to acetic acid, 2-methyl-1-butanol, 2-pentylfuran, and 6-PP was 0.007, 0.007, 0.006, and 0.005 U mL^−1^, respectively, significantly higher than the control (0.002 U mL^−1^). The β-1,3-glucanase activity found in *A. thaliana* after exposure to acetic acid, 2-methyl-1-butanol, 2-pentylfuran, and 6-PP was 0.213, 0.262, 0.222, and 0.222 U mL^−1^, respectively, significantly higher than in the control (0.086 U mL^−1^).

**Figure 9:**
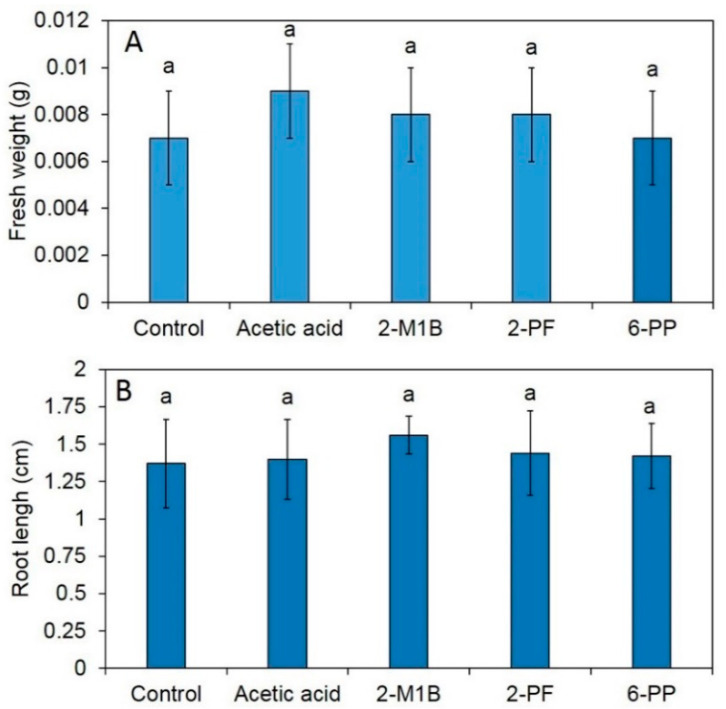
Effects of volatile organic compounds on the phenotypic characteristics of fresh weight (**A**) and root length (**B**) of *A. thaliana*. Different letters indicate statistically significant differences among treatments (*p* < 0.05) using the Tukey’s test.

The effects of each commercial volatile compound on the plant phenotype (fresh weight and root length) in *A. thaliana* are shown in Figure 9. The results show no statistically significant differences among the treated and untreated samples (Figure 9).

## 4. Discussion

Several fungal volatiles emitted by *Trichoderma* species have recently been reported to limit fungal growth [1,4,37]. In the present study, *T. asperelloides* PSU-P1 produced and emitted a complex mix of VOCs to suppress fungal pathogens (Figure 2). The SPME trapped the VOCs from *T. asperelloides* PSU-P1 and GM/MS were tentatively identified as acetic acid, 2-pentyl furan, 2-methyl-1-butanol, and 6-PP (Figure 8). Among the four compounds found in this study, three have been reported to show antifungal activity against several fungal pathogens; namely, acetic acid [38], 2-methyl-1-butanol [39], and 6-PP [4,40]. We tested the antifungal abilities of commercial compounds on the fungal growth of plant pathogens (Table 4), and we found that 6-PP was the most effective volatile against the plant pathogens *Ganoderma* sp., *P. oxalicum*, *S. rolfsii,* and *S. cucurbitacearum* (Table 4).

Among the VOCs produced by *Trichoderma* species, 6-PP with a sweet coconut-like aroma was considered the most important produced by *T. asperellum* [4], *T. viride* [41], *T. harzianum* [42], *T. koningii* [43], and *T. atroviride* [44,45]. In this study we found *T. asperelloides* also produces 6-PP (Figure 8). 6-PP and its analogs have shown antifungal activity against plant pathogens [6,35]. However, herein, the antifungal ability of each commercial compound was lower than of the VOCs emitted by *T. asperelloides* PSU-P1. This phenomenon may be because some compounds might work together synergistically to inhibit the fungal growth of pathogens (Figure 2). It is presumed that in PDA cultures, *Trichoderma* produces other metabolites than those four compounds described in this study. Therefore, a different effect of the individual metabolites and their entire spectrum on plants may be observed.

It has been shown that VOCs from some *Trichoderma* species promote plant growth [4,46,47]. For instance, VOCs from *T. pseudokoningii* and *T. viride* have been shown to increase *Arabidopsis* growth and tomato plant biomass, respectively [47]. In addition, Jalali et al. [48] showed that VOCs from *T. koningii* induce growth promotion in *A. thaliana*. Furthermore, the VOCs of *T. asperellum* T1 have recently been shown to increase plant growth in lettuce [4]. In this study, *T. asperelloides* PSU-P1 emitted plant growth-promoting VOCs increasing fresh weigh, root length, and total chlorophyll content in *A. thaliana* (Figure 3 and Figure 4), in agreement with recent reports [4,48]. Among the found compounds found in this study, two have been reported to promote plant growth; namely, 2-pentylfuran [49] and 6-PP [4,48]. However, we tested the effects of commercial compounds on plant growth in *A. thalaiana* (Figure 8 and Figure 9), and the results showed no significant differences from the control. A VOC mixture could potentially have such effects, while each volatile alone does not affect plant growth in *A. thaliana*. To increase the plant biomass, several factors are involved in the closed atmosphere such as the presence of the fungal culture in the treatment plate can be responsible for a significant difference in relative humidity and CO_2_ exchange that, influencing the leaf metabolism could induce a higher growth of plant tissues respect to the control. However, we did not investigate this phenomenon due to the complicated experimental setup.

The role of VOCs on the activation of defense responses has been reported in several plants [16,50]. Naznin et al. [36] showed that the VOCs emitted by plant growth-promoting fungi induce a high expression of defense-related genes, i.e., SA-responsive gene *PR1* and JA-responsive gene *PDF1.2* in *A. thaliana*. The *Trichoderma* VOCs affected the gene expression involved in defense responses in *A. thaliana* [46]. The results from our study showed that the expression of *CHI*, *GLU*, and *POD* was significantly higher than in the control (Figure 5). This is in agreement with Kim et al. [51], who showed that VOCs from *Bacillus* sp. JS cause the upregulation of *PR-2* encoding β-1,3-glucanase and of PR-3 encoding chitinase. Furthermore, Jain et al. [52] demonstrated that the microbial VOCs released by *Bacillus* sp. strain SJ-5 induce defense-related genes, lipoxygenase, phenylalanine ammonia-lyase 2, peroxidase, polyphenol oxidase, endo-1,3-beta-glucanase, catalase, defensin-like protein, vegetative storage protein, and chitinase class I in *Glycine max*. Therefore, the upregulation of *CHI*, *GLU*, and *POD* genes in *A. thaliana* may contribute to plant resistance by the production of defense-related enzymes against fungal pathogens.

Based on the results from our study, the VOCs from *T. asperelloides* PSU-P1 increased the activity of defense-related enzymes (*POD*) and cell wall-degrading enzymes (chitinase and β-1,3-glucanase), as shown in Figure 6. The application of 6-Pentyl-alpha-pyrone (6PAP), a volatile compound from *T. harzianum,* enhanced the activity of *POD*, polyphenol oxidase, and β-1,3-glucanase in maize against seedling blight caused by *Fusarium moniliforme* [50]. However, we investigated the effect of VOCs on the plants in this study, and the influence of pathogens was not observed in this study. The treatment with VOCs from *T. asperellum* T1 in lettuce induced high activity of chitinase and β-1,3-glucanase [4]. Furthermore, we tested the effects of each commercial volatile compound (i.e., acetic acid, 2-methyl-1-butanol, 2-pentylfuran, and 6-PP) on the induction of defense responses in *A. thaliana* (Figure 9). We found that all four compounds were able to induce the activity of POD, chitinase, and β-1,3-glucanase in treated *A. thaliana*. The application of 6-PP to induce defense responses in lettuce has been reported recently [4]. We, therefore, identified new inducers of defense responses from *T. asperelloides* PSU-P1 in *A. thaliana*.

Several reports have investigated the effect of VOCs emitted from *Trichoderma* species on plant metabolism. For instance, Kottb [53] showed that the VOCs from *T. asperellum* IsmT5 increase trichome numbers, accumulate defense-related compounds, and increase the expression of defense-related genes. Wonglom [5] demonstrated that the VOCs emitted by *T. asperellum* are involved in antifungal activity, defense responses, and promotion of plant growth in lettuce (*Lactuca sativa*). The results from this study demonstrated that the VOCs emitted from *T. asperelloides* PSU-P1 increased plant growth and the enzyme activity of defense-related enzymes and increased the expression of defense-related genes in agreement with Kottb [53]. However, an effect of VOCs on the change in plant physiology was not observed in this study.

## 5. Conclusions

Herein, we described the possible mechanisms of the VOCs emitted by *T. asperelloides* PSU-P1 involved in antifungal activity against fungal pathogens’ defense responses by upregulating CHI and GLU, enhanced cell wall-degrading enzyme activity, and increased growth of *A. thaliana*. However, the results obtained in this work highlight the effect of VOCs and some individual volatiles on plant growth and plant defense responses. In order to better understand the role of VOCs for bio-fumigant, the effect of VOCs on the suppression of fungal pathogens and their field application need to be conducted in the near future.

## Figures and Tables

**Table 1:** List of the microorganisms used in this study.

Microorganism		Disease	Reference
Antagonist	Fungal Pathogen		
*Trichoderma asperelloides* PSU-P1	–	–	[18]
	*Colletotrichum* sp.	Leaf blight	CCPM
	*Corynespora cassiicola*	Leaf spot	[19]
	*Curvularia lunata*	Leaf spot	[20]
	*Ganoderma* sp.	Basal stem rot	CCPM
	*Penicillium oxalicum*	Blue mold	[21]
	*Neopestalotiopsis clavispora*	Flower blight	[22]
	*Sclerotium rolfsii*	Southern blight	[23]
	*Stagonosporopsis cucurbitacearum*	Gummy stem blight	[24]

CCPM, Culture Collection of Pest Management.

**Table 2:** Specific primer pairs for the gene expression determinations with quantitative real-time reverse transcription polymerase chain reaction (qRT-PCR).

Gene	Accession Number	Primer	Sequence (5′ → 3′)	Product Size (bp)
*ACT*	NM001036427	ACT-F	CTCCCATTCCCTTCTCCTTC	247
		ACT-R	CGAGGACGACCCACAATACT	
*CHI*	NM129919	Chi-F	TAGCTTCGGTGCTTCCATCT	159
		Chi-R	GCACATGGGAACTCTGGTTT	
*GUL*	NM115586	Glu-F	TGGTGTCAGATTCCGGTACA	192
		Glu-R	TCATCCCTGAACCTTCCTTG	
*POD*	BT001238	POD-F	ACCAACAGACCAGACCCAAG	244
		POD-R	CGAACGTGTTGCTGCTGTAT	

**Table 3:** International Union of Pure and Applied Chemistry (IUPAC) names of the volatile compounds produced by *T. asperelloides* PSU-P1 identified through SPME GC/MS analysis.

RT (min)	Volatile Compound	*m*/*z*	Formula	Similarity	Total Area
10.93	2-methyl-1-butanol *	88	C_5_H_12_O	95	6.96
11.34	2-pentyl furan	138	C_9_H_14_O	96	30.59
17.98	Acetic acid	60	C_2_H_4_O_2_	97	1.96
34.71	6-penthyl-2H-pyran-2-one	108	C_10_H_14_O_2_	97	5.45

* The results from three replicates. SPME, solid phase microextraction; RT, retention time.

**Table 4:** Antifungal activities of commercial volatile compounds against fungal pathogens.

Fungal Pathogen	Disease	Antifungal Ability ^1^			
		Acetic Acid	2-PF	2M1B	6-PP
*Colletotrichum* sp.	Leaf blight	−	−	−	−
*C. cassiicola*	Leaf spot	−	−	−	−
*C. lunata*	Leaf spot	−	−	−	−
*Ganoderma* sp.	Basal stem rot	+	+	+	+
*P. oxalicum*	Blue mold	−	+	+	+
*N. clavispora*	flower blight	−	−	−	−
*S. rolfsii*	Southern blight	−	−	−	+
*S. cucurbitacearum*	Gummy stem blight	−	−	−	+

^1^ The experiment was conducted by volatile antifungal bioassay in 9 cm Petri dish. 2PF, 2-pentyl furan; 2M1B, 2-methyl-1-butanol; 6-PP, 6-pentyl-2H-pyran-2-one. “+” antifungal activity of the VOCs inhibits fungal growth; “−” no antifungal activity of the VOCs.
